# Case series of delusional parasitosis in an emergency department: Sociodemographic features and clinical outcomes

**DOI:** 10.1192/j.eurpsy.2021.1384

**Published:** 2021-08-13

**Authors:** A. Guàrdia, A. González-Rodríguez, M. Betriu, F. Estrada, M.V. Seeman, I. Parra Uribe, J. Labad, D. Palao Vidal

**Affiliations:** 1 Mental Health, Parc Taulí University Hospital. Autonomous University of Barcelona (UAB). I3PT, Sabadell, Spain; 2 Department Of Mental Health., Parc Tauli University Hospital. Sabadell, Barcelona (Spain), Sabadell, Spain; 3 Psychiatry, University of Toronto, Toronto, Canada; 4 Mental Health, Hospital of Mataró. Consorci Sanitari del Maresme. CIBERSAM., Mataró, Spain; 5 Mental Health, Parc Taulí University Hospital. Autonomous University of Barcelona (UAB). I3PT. CIBERSAM, Sabadell, Spain

**Keywords:** Delusional parasitosis, Emergency department, Delusional disorder, Ekbom syndrome

## Abstract

**Introduction:**

A delusion of parasitosis is defined as the fixed, false belief of infestation by invisible organisms or fibrous material of unknown origin. The differential diagnosis is true infection, substance use disorder, dementia or other neuropsychiatric disease.

**Objectives:**

Our goal was to characterize delusions of parasitosis, classically named Ekbom syndrome, among individuals attending our emergency department (ED).

**Methods:**

Over a four-year period (2017-2020), we carried out a retrospective case-register study of patients with DSM-5 Ekbom syndrome attending an ED that provides mental health services to an area of nearly 450.000 inhabitants in Sabadell (Barcelona, Spain).

**Results:**

There were 13 eligible patients: 7 were diagnosed for the first time and 6 had multiple episodes. Female-to-male ratio was 1.6:1; average age was 56.9. The most common diagnosis was delusional disorder (n=5;8.5%), followed by schizophrenia (n=3;23.1%) and organic disorders (n=2;15.4%). Origin: Africa (n=5;38.5%), South-America (n=4;30.8%) and Spain (n=4;30.8%). Fifty percent showed poor treatment compliance. Antipsychotics used: risperidone (n=8;61.54%), olanzapine (n=4;30.8%). Five patients received antidepressants. Most patients had previously been seen by other medical specialties (internal medicine, dermatology and hematology). ‘’Match box sign’’: 7 patients (53.8%). Cerebral atrophy was present on brain scan in 4 patients. After discharge: acute psychiatric unit (n=7), outpatient appointments (n=4), day hospital (n=1) and 1 to a psychogeriatric unit.

**Conclusions:**

Delusions of parasitosis are rare in our emergency department. The typical patient is a postmenopausal woman, a visitor or immigrant to Spain. Effective treatment requires a focus on cultural, gender, and age aspects, with close cooperation between psychiatry and other relevant specialties.

